# Plasma and CSF neurofilament light chain distinguish neurodegenerative from primary psychiatric conditions in a clinical setting

**DOI:** 10.1002/alz.14278

**Published:** 2024-10-06

**Authors:** Dhamidhu Eratne, Matthew J. Y. Kang, Courtney Lewis, Christa Dang, Charles B. Malpas, Michael Keem, Jasleen Grewal, Vladimir Marinov, Amy Coe, Cath Kaylor‐Hughes, Thomas Borchard, Chhoa Keng‐Hong, Alexandra Waxmann, Burcu Saglam, Tomas Kalincik, Richard Kanaan, Wendy Kelso, Andrew Evans, Sarah Farrand, Samantha Loi, Mark Walterfang, Christiane Stehmann, Qiao‐Xin Li, Steven Collins, Colin L. Masters, Alexander F. Santillo, Henrik Zetterberg, Kaj Blennow, Samuel F. Berkovic, Dennis Velakoulis, The MiND Study Group

**Affiliations:** ^1^ Neuropsychiatry Centre The Royal Melbourne Hospital Melbourne Victoria Australia; ^2^ Department of Psychiatry The University of Melbourne Melbourne Victoria Australia; ^3^ The Florey Melbourne Victoria Australia; ^4^ Institute of Health and Wellbeing Federation University Melbourne Victoria Australia; ^5^ National Ageing Research Institute Melbourne Victoria Australia; ^6^ Department of General Practice The University of Melbourne Melbourne Victoria Australia; ^7^ Department of Medicine (Royal Melbourne Hospital) University of Melbourne Melbourne Victoria Australia; ^8^ Melbourne School of Psychological Sciences University of Melbourne Melbourne Victoria Australia; ^9^ School of Rural Health Monash University Melbourne Victoria Australia; ^10^ Melbourne School of Population & Global Health and Department of Medical Education University of Melbourne Melbourne Victoria Australia; ^11^ Western Health St. Albans Victoria Australia; ^12^ Mildura Base Public Hospital Melbourne Victoria Australia; ^13^ Alfred Mental and Addiction Health Alfred Health Melbourne Victoria Australia; ^14^ Ramsay Clinic Northside Frederick St Sydney Australia; ^15^ Department of Psychiatry & Mental Health Sarawak General Hospital Kuching Sarawak Malaysia; ^16^ Neuroimmunology Centre Department of Neurology Royal Melbourne Hospital Melbourne Victoria Australia; ^17^ CORe Department of Medicine University of Melbourne Melbourne Victoria Australia; ^18^ Department of Psychiatry The University of Melbourne Austin Hospital Melbourne Victoria Australia; ^19^ Department of Clinical Sciences Clinical Memory Research Unit Faculty of Medicine Lund University Malmö Sweden; ^20^ Department of Psychiatry and Neurochemistry Institute of Neuroscience and Physiology the Sahlgrenska Academy at the University of Gothenburg Mölndal Sweden; ^21^ Clinical Neurochemistry Laboratory Sahlgrenska University Hospital Mölndal Sweden; ^22^ Department of Neurodegenerative Disease UCL Institute of Neurology London UK; ^23^ UK Dementia Research Institute at UCL London UK; ^24^ Hong Kong Center for Neurodegenerative Diseases Hong Kong China; ^25^ Wisconsin Alzheimer's Disease Research Center University of Wisconsin School of Medicine and Public Health University of Wisconsin‐Madison Madison Wisconsin USA; ^26^ Institute of Neuroscience and Physiology University of Gothenburg Mölndal Sweden; ^27^ Clinical Neurochemistry Lab Sahlgrenska University Hospital Mölndal Sweden; ^28^ Paris Brain Institute ICM Pitié‐Salpêtrière Hospital Sorbonne University Paris France; ^29^ Neurodegenerative Disorder Research Center Division of Life Sciences and Medicine and Department of Neurology Institute on Aging and Brain Disorders University of Science and Technology of China and First Affiliated Hospital of USTC Hefei Anhui P.R. China; ^30^ Epilepsy Research Centre Department of Medicine Austin Health The University of Melbourne Melbourne Victoria Australia

**Keywords:** biomarkers, dementia, diagnosis, neurofilament light chain protein, psychiatric disorders

## Abstract

**INTRODUCTION:**

People with neurodegenerative disorders (ND) frequently face diagnostic delay and misdiagnosis. We investigated blood and cerebrospinal fluid (CSF) neurofilament light chain (NfL) to distinguish ND from primary psychiatric disorders (PPD), a common challenge in clinical settings.

**METHODS:**

Plasma and CSF NfL levels were measured and compared between groups, adjusting for age, sex, and weight.

**RESULTS:**

A total of 337 participants were included: 136 ND, 77 PPD, and 124 Controls. Plasma NfL was 2.5‐fold elevated in ND compared to PPD and had strong diagnostic performance (area under the curve, [AUC]: 0.86, 81%/85% specificity/sensitivity) that was comparable to CSF NfL (2‐fold elevated, AUC: 0.89, 95%/71% specificity/sensitivity). Diagnostic performance was especially strong in younger people (40– < 60 years). Additional findings were cutoffs optimized for sensitivity and specificity, and issues important for future clinical translation.

**CONCLUSIONS:**

This study adds important evidence for a simple blood‐based biomarker to assist as a screening test for neurodegeneration and distinction from PPD, in clinical settings.

**Highlights:**

- NfL levels were significantly higher in ND versus PPD.
- Plasma NfL showed strong diagnostic performance, comparable to CSF NfL, to distinguish ND from PPD.
- Diagnostic performance was higher in younger people, where diagnostic challenges are greater.
- Further research is needed on analytical and reference range factors, for clinical translation.
- These findings support a simple screening blood test for neurodegeneration.

## 1 INTRODUCTION

Despite major improvements in clinical assessment, many patients with neurodegenerative disorders (ND) still face significant barriers and challenges to timely, accurate diagnosis; delays often last several years even with gold‐standard assessments.^1^ These challenges are worse for younger people (onset of symptoms < 60–65 years of age), where a wider range of ND and less typical presentations of ND are more common, and substantial overlap in symptoms with psychiatric disorders exists.^1^, ^2^, ^3^ Methods to distinguish ND from primary psychiatric disorders (PPD) are a major unmet need.

One of the most well‐established biomarkers for neuronal injury, neurofilament light chain protein (NfL), has shown great promise in distinguishing broad causes of ND from PPD and non‐neurodegenerative disorders. We have previously demonstrated the strong diagnostic utility of cerebrospinal fluid (CSF) NfL^3^, ^4^, ^5^, ^6^, ^7^; however, a blood‐based biomarker could be a less invasive, more easily accessible option to improve diagnosis, which is critical for improved outcomes for patients, families, healthcare systems, and clinical trials.

Research investigating blood NfL in distinguishing diverse ND directly from diverse PPD in clinical settings, especially in younger populations, has been limited. Most studies have focused on comparing NfL levels between different NDs and controls^8^, ^9^, ^10^, ^11^, ^12^, ^13^ Some studies have investigated CSF and blood NfL for broad ND diagnosis in clinical settings, finding elevated concentrations of NfL in ND, diagnostic utility, and/or increasing diagnostic certainty.^14^, ^15^, ^16^, ^17^, ^18^, ^19^, ^20^ The latter studies have primarily been in older people in memory clinic settings, and most have not specifically been compared to diverse PPD. Studies that have included PPD have either been small (e.g., *n* = 17),^20^ or grouped PPD with healthy controls or “non‐neurodegenerative disorder” or similar categorizations, and did not specifically compare ND to PPD,^14^ despite some evidence that plasma NfL may be mildly elevated in PPD compared to healthy controls.^21^, ^22^ While blood NfL has shown strong diagnostic performance in distinguishing PPD from specific ND subtypes, such as behavioral variant frontotemporal dementia (bvFTD),^8^, ^9^, ^21^, ^22^, ^23^, ^24^ broader comparative studies across diverse ND and PPD populations are needed to fully realize its clinical utility and for real‐world clinical translation. Finally, there is also increasing recognition of the importance of further research in clinical settings, including important covariates that can influence individual levels and reference ranges and interpretations, such as age, analytical platform, and technological factors, for standardization and proper clinical translation.^25^, ^26^, ^27^, ^28^, ^29^

This study aims to address the significant gaps in research by comparing diverse ND and PPD reflective of real‐world practice, with a focus on younger populations where the diagnostic overlap between ND and PPD is particularly challenging, aiming to provide a more nuanced understanding of NfL's diagnostic utility in a clinical setting. The primary aim of this study was to investigate differences in blood and CSF NfL concentrations between ND and PPD seen in a clinical neuropsychiatry service, and their diagnostic performance in differentiating between the two diagnostic groups (Aim 1). We also aimed to investigate the accuracy and utility of age‐based cutoff levels/concentrations between younger and older patients and cutoffs optimizing sensitivity and specificity (Aim 2). For issues related to clinical translation, we also aimed to perform exploratory analyses to compare the accuracy of different classification systems, including our previously described age‐adjusted percentile and *z*‐score models,^22^ and previously described reference ranges (Aim 3).

## 2 METHODS

### 2.1 Study cohorts

This study included participants prospectively recruited between June 2019 and April 2023, who had provided a blood sample for NfL analysis. A subset of patients had CSF collected for clinical purposes, with remnant samples available for NfL analysis. The patient cohorts were people referred for diagnostic assessment and management of possible ND to the Neuropsychiatry Centre at The Royal Melbourne Hospital, a quaternary service receiving referrals for diagnostically complex cases from primary care and other specialist services within Australia. Patients received comprehensive multidisciplinary assessments and multimodal investigations, including CSF analysis, with gold standard consensus diagnosis based on established diagnostic criteria, as previously described in detail.^3^, ^5^ Control participants were people recruited from the community, with no symptoms or diagnoses of neurological or neurodegenerative disorders, no active psychiatric symptoms or conditions.

This study included 38 patients (26 ND, 12 PPD) from our previous CSF study.^3^ The remaining patients (*n* = 175), controls (*n* = 124), and data in this study (including all blood NfL data), have not been described previously. Diagnostic group categorization was determined based on the most recent diagnosis at longitudinal follow‐up, blinded to NfL levels, as previously described.^3^, ^5^

CSF and EDTA plasma samples were stored at ‐80°C. Plasma NfL was measured using NF‐Light kits on a Quanterix Single molecule array (Simoa) HD‐X analyzer, according to the manufacturer's instructions (Quanterix Corporation, Billerica, MA, USA). CSF NfL was measured using a commercial enzyme‐linked immunosorbent assay (ELISA; NF‐light; UmanDiagnostics, Sweden).

**RESEARCH IN CONTEXT**

1. **Systematic review**: We reviewed the literature on PubMed on neurofilament light (NfL). There is extensive data on cerebrospinal fluid (CSF) and blood NfL in neurodegenerative disorders (ND), including recent comprehensive reviews. However, there is limited literature on blood NfL in primary psychiatric disorders (PPD), specifically on blood NfL to assist with the common clinical diagnostic challenge of distinguishing diverse ND from PPD.
2. **Interpretations**: This study demonstrated the strong diagnostic performance of blood NfL, comparable to CSF NfL, adding important evidence on the strong diagnostic utility of blood NfL to assist the clinical distinction of ND from PPD, especially in younger people, in real‐world clinical settings.
3. **Future directions**: Our ongoing and future studies aim to replicate these findings in larger cohorts and from more settings including primary care settings, as well as further investigating and addressing important issues still required for clinical translation and the possibility of a simple, widely available blood test to reduce diagnostic delay and misdiagnosis, and dramatically improve outcomes for patients with cognitive and psychiatric symptoms, their families, clinical trials, and healthcare systems.

### 2.2 Statistical analyses

Statistical analyses were performed using R version 4.3.2 (2023‐10‐31). As several biomarker distributions were non‐Gaussian even when log‐transformed, biomarker levels in different groups were compared using standardized bootstrapped quantile regression, with age and sex as additional covariates. To examine statistical effects, standardized quantile regression coefficients (ß) were interpreted along with 95% confidence intervals (CIs). Receiver operating characteristic (ROC) curve analyses were performed to investigate diagnostic utility between different combinations of groups. Bootstrapped DeLong test was used to compare ROC curves. Optimal cutoffs were selected based on Youden's J, and alternative cutoffs for screening based on optimized specificity and sensitivity. Additional diagnostic test parameters were computed: positive and negative likelihood ratios, positive and negative predictive values, overall accuracy, and diagnostic odds ratio (DOR). DOR is a single indicator of test performance that combines the sensitivity and specificity of a diagnostic test, reflecting the odds of a positive test result in patients with ND relative to the odds of a positive test result in those without. A higher DOR indicates better discriminatory test performance, with a DOR of 1 indicating that the test does not discriminate between patients with and without ND. Additional sensitivity analyses were performed: excluding extreme outliers and performing all quantile regressions with weight included as a covariate. As the results were similar, the results excluding weight were presented to maximize the sample sizes for analyses and presented results (since not all participants had weight data).

This study, part of The Markers in Neuropsychiatric Disorders Study (The MiND Study, https://themindstudy.org), was approved by the Human Research Ethics Committee at Melbourne Health (2016.038, 2017.090, 2018.371, 2020.142).

## 3 RESULTS

### 3.1 Study cohort

The total study cohort included 337 participants: 136 with ND, 77 with PPD, and 124 Controls (Table 1). Controls were slightly older at 63.2 years compared to the other groups (ND 60.8 years, PPD 54.8 years) and had a greater proportion of females (71% compared to ND 43%, PPD 51%). 250 people had weight data. PPD had a higher weight (84 kg) compared to ND (75 kg) and Controls (76 kg).

**TABLE 1:** Study demographics and plasma and CSF NfL levels in ND, PPD, and Controls.

	ND	PPD	Control	
Parameter	*N = 136*	*N = 77*	*N = 124*	*N*
Age	60.8 (55.9;65.9)	54.8 (46.6;61.8)	63.2 (56.0;70.0)	337
Sex (female)	59 (43.4%)	39 (50.6%)	88 (71.0%)	337
Weight	75.0 (59.8;89.1)	84.0 (73.4;98.8)	76.0 (66.0;84.0)	250
	(*n* = 102)	(*n* = 58)	(*n* = 90)	
Plasma NfL (pg/mL)	25.2 (15.8;39.6)	10.1 (7.85;12.5)	12.5 (8.70;17.8)	337
CSF NfL (pg/mL)	1048 (741;1585)	495 (386;671)	–	85
	(*n* = 63)	(*n* = 22)		

*Note*: Data are median [interquartile range] or *n* (%).

Abbreviations: CSF, cerebrospinal fluid; NfL, neurofilament light chain; ND, neurodegenerative disorder; PPD, primary psychiatric disorder.

All 337 people had plasma NfL. Both plasma and CSF NfL concentrations were determined for 84 people (plasma+CSF group: 63 ND, 22 PPD), the remainder had only plasma NfL data available (plasma‐only group: 252 people, ND 73, PPD 55, 124 Controls). Details of these subsets can be found in Tables S1 and S2.

The ND group consisted of Alzheimer's disease (AD, *n* = 44), bvFTD, *n* = 16, Lewy body dementia (DLB, *n* = 7), dementia not‐otherwise‐specified (dementia NOS, *n* = 9), Huntington's disease (*n* = 17), vascular dementia (*n* = 6), mixed Alzheimer's/vascular dementia (*n* = 3), Creutzfeldt–Jakob disease (*n* = 2), substance‐induced dementia (*n* = 2), and Other ND (*n* = 30, which included autoimmune encephalitis, cerebral amyloid angiopathy, corticobasal syndrome, CNS vasculitis, Down syndrome, Fahr disease, metabolic disorders, Niemann–Pick Type C, Parkinson's disease, and cerebellar degenerative disorder). The PPD group consisted of major depressive disorder (MDD, *n* = 23), schizophrenia spectrum disorders (*n* = 19), functional neurological/cognitive disorders (*n* = 9), bipolar affective disorder (BPAD, *n* = 6), and Other PPD (*n* = 18, including anxiety disorders [*n* = 6], post‐traumatic stress disorders [*n* = 4], obsessive‐compulsive disorders [*n* = 4], personality disorders [*n* = 2], bvFTD “phenocopy syndrome” [*n* = 2], undifferentiated psychiatric disorders [*n* = 2]).

### 3.2 Aim 1: Plasma and CSF NfL levels and diagnostic utility in neurodegenerative and PPD

#### 3.2.1 In a subset of patients with both CSF and plasma samples, compared to the subset with only plasma

We first separately analyzed the subset of patients with both CSF and plasma levels (plasma+CSF), and the subset with only plasma levels (plasma‐only), to determine comparability, and thus determine whether the total cohort could be analyzed as a whole.

For the plasma+CSF group (*n* = 85, 63 ND, 22 PPD), NfL levels were significantly elevated in ND compared to PPD, in CSF (median 1048 pg/mL vs. 495 pg/mL, standardized quantile regression coefficient ß: 0.09, 95% CI: [0.04, 0.53], *p* < 0.001), and in plasma (median 24.4 pg/mL vs. 10.3 pg/mL, ß: 0.07, 95% CI: [0.03, 0.54], *p* < 0.001), Table S1.

The plasma‐only group (*n* = 128, 73 ND, 55 PPD) had comparable results, with plasma NfL significantly elevated in ND compared to PPD (median 26.8 pg/mL vs. 10.1 pg/mL, ß: 0.18, 95% CI: [0.08, 0.70], *p* < 0.001), Table S2. We therefore analyzed the entire cohort and presented detailed results below, with a focus on plasma NfL.

#### 3.2.2 In entire cohort

**FIGURE 1:**
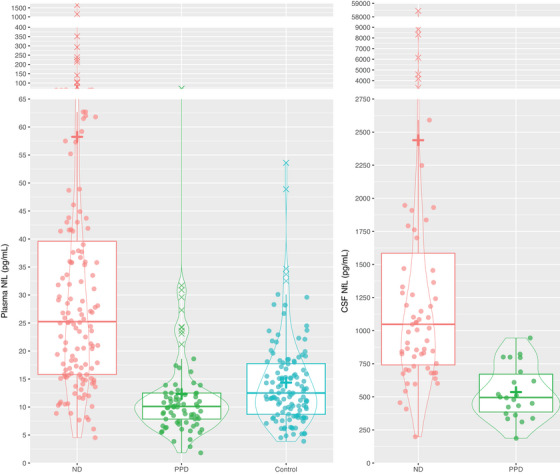
Plasma and CSF NfL levels in ND, PPD, and Controls. +, mean level. CSF, cerebrospinal fluid; NfL, neurofilament light chain; ND, neurodegenerative disorder; PPD, primary psychiatric disorder.

NfL levels were significantly elevated in ND compared to PPD, 2.5‐fold in plasma (median 25.2 pg/mL vs. 10.1 pg/mL; ß: 0.10 [0.05, 0.43], *p* < 0.001), and 2‐fold in CSF (median 1048 pg/mL vs. 495 pg/mL, ß: 0.09, 95% CI: [0.04, 0.53], *p* < 0.001), Table 1 and Figure 1.

Compared to controls, plasma NfL levels were higher in ND (median 25.2 pg/mL vs. 12.5 pg/mL, ß: 0.13, 95% CI: [0.07, 0.48], *p* < 0.001), but there was no difference between PPD and controls (10.1 pg/mL vs. 12.5 pg/mL, ß: 0.00 [‐0.03, 0.03], *p* = 0.998).

**FIGURE 2:**
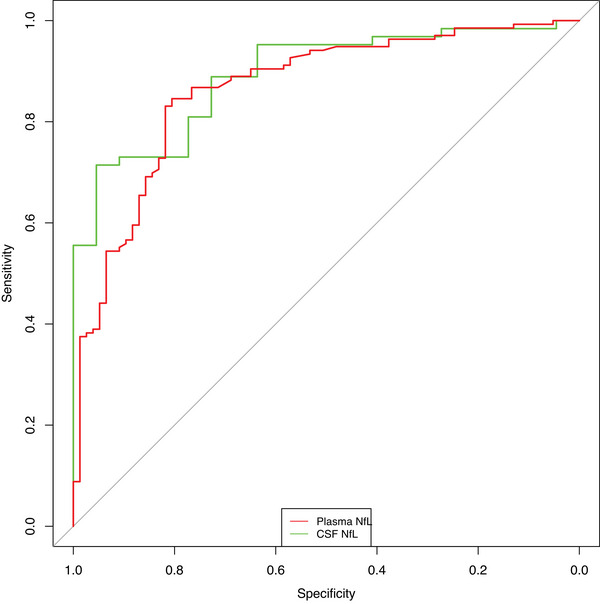
Receiver operator characteristic analysis curves for plasma and CSF NfL. CSF, cerebrospinal fluid; NfL, neurofilament light chain.

ROC curve analyses (Figure 2, Table 2) demonstrated strong diagnostic performance of plasma NfL in distinguishing ND from PPD, with an area under the curve (AUC) of 0.86 [0.81, 0.92], with an optimal cutoff of 14.1 pg/mL associated with 81% specificity, 85% sensitivity, 4.34 positive likelihood ratio (LR+), 0.19 negative likelihood ratio (LR‐), 88% positive predictive value (PPV), 75% negative predictive value (NPV), 22.6 DOR, 83% accuracy (base rate/disease prevalence 63.85%).

**TABLE 2 alz14278-tbl-0002:** Details of ROC curve analyses and diagnostic test parameters.

Categorization	Age	AUC	Cutoff	Spec	Sens	LR±	LR‐	PPV	NPV	DOR	Accuracy
**ND vs. PPD**											
Plasma NfL	All	0.86 (0.81, 0.92)	14.1	81%	85%	4.34	0.19	88%	75%	22.63	83%
CSF NfL	All	0.89 (0.82, 0.96)	823 (532)	95% (64%)	71% (95%)	15.71 (2.62)	0.30 (0.07)	98% (88%)	54% (82%)	52.5 (35)	78% (87%)
Plasma NfL	Younger 40– < 60	0.89 (0.83, 0.96)	14.6^a^	90%	84%	8.06	0.18	89%	84%	45.15	87%
CSF NfL	Younger 40– < 60	0.97 (0.92, 1)	814^b^ (558)	100% (85%)	88% (96%)	^g^(6.23)	0.13 (0.05)	100% (92%)	81% (92%)	^g^(126.5)	92% (92%)
Plasma NfL	Older 60– < 70	0.76 (0.63, 0.89)	11.9^c^	55%	91%	2.03	0.16	85%	69%	12.71	82%
CSF NfL	Older 60– < 70	0.76 (0.59, 0.92)	967^d^	100%	55%	^g^	0.45	100%	36%	^g^	64%
**AD vs. PPD** ^e^											
Plasma NfL	All	0.89 (0.84, 0.95)	14.6	82%	93%	5.13	0.08	75%	95%	61.50	86%
CSF NfL	All	0.95 (0.90, 1)	824	95%	87%	19.07	0.14	96%	84%	136.5	90%
**bvFTD vs. PPD** ^f^											
Plasma NfL	All	0.79 (0.65, 0.92)	11.9	69%	81%	2.61	0.27	35%	95%	9.57	71%
CSF NfL	All	0.86 (0.70, 1)	975	100%	63%	^g^	0.38	100%	88%	^g^	90%
**Previous reference ranges/cutoffs**											
**ND vs. PPD**											
*z*‐score	All	0.80 (0.73, 0.86)	1.44	74%	74%	2.83	0.36	83%	61%	7.92	74%
	Younger 40– < 60	0.87 (0.80, 0.95)	1.70	85%	82%	5.62	0.21	85%	82%	26.68	84%
	Older 60– < 70	0.76 (0.64, 0.89)	0.37	55%	91%	2.03	0.16	85%	69%	12.71	82%
95th percentile	All ages			77%	68%	2.89	0.42	84%	57%	6.9	71%
	40– < 60			83%	82%	4.92	0.22	84%	82%	22.8	83%
	60– < 70			65%	63%	1.80	0.57	84%	38%	3.2	64%
95th percentile adjusted levels	All			88%	45%	3.84	0.62	87%	48%	6.1	61%
	40– < 60			92%	54%	6.48	0.50	87%	66%	12.9	72%
	60– < 70			90%	42%	4.21	0.64	92%	35%	6.5	55%
Simrén et al. plasma cutoffs	All		Age‐based	73%	71%	2.59	0.4	82%	58%	6.4	71%
Simrén et al. with adjusted NfL levels	All		Age‐based	90%	46%	4.39	0.61	89%	48%	7.2	62%
Kang et al. CSF cutoffs	All		Age‐based	73%	83%	3.03	0.24	90%	59%	12.6	80%
Eratne et al. CSF cutoff	All		582	64%	94%	2.58	0.10	88%	86%	25.8	86%

Abbreviations: AD, Alzheimer's disease; AUC, the area under the curve; bvFTD, behavioural variant frontotemporal dementia; CSF, cerebrospinal fluid; LR+, positive likelihood ratio; LR‐, negative likelihood ratio; NfL, neurofilament light chain; ND, neurodegenerative disorder; NPV, negative predictive value; PPD, primary psychiatric disorder; PPV, positive predictive value; Sens, sensitivity; Spec, specificity.

^a^ Alternative cutoffs optimizing specificity were: 30.8 pg/mL (100% specificity), 24.4 pg/mL (98% specificity), 24 pg/mL (96% specificity), 14.6 pg/mL (90%), and for sensitivity: 6.0 pg/mL (100%), 8.0 pg/mL (98%), 10.1 pg/mL (94%), 11.5 pg/mL (90%).

^b^ Alternative CSF cutoffs for specificity were 814 pg/mL (100% specificity), 743 pg/mL (92%), 558 pg/mL (85%), and for sensitivity were 445 pg/mL (100% sensitivity), 558 pg/mL (96% sensitivity, 85% specificity), 638 pg/mL (92%).

^c^ Alternative cutoffs associated with 100%, 95%, and 90% specificity were 74.9 pg/mL, 31.8 pg/mL, and 31 pg/mL, respectively. Alternative cutoffs optimizing for sensitivity were 7.6 pg/mL (100% sensitivity), 10.4 pg/mL (98%), 10.85 pg/mL (95%), and 11.9 pg/mL (91%).

^d^ Alternative cutoffs optimizing for specificity were 967 pg/mL (100%) and 823 pg/mL (88%), and for sensitivity were 511 pg/mL (97%), 571 pg/mL (94%), 600 pg/mL (90%).

^e^ Higher DOR and accuracy in younger people (154 and infinity, 91% and 96%, for plasma and CSF NfL respectively).

^f^ Higher DOR and accuracy in younger people (16.5 and infinity, 86% and 94%, for plasma and CSF NfL respectively).

^g^ Value of infinity/not able to be calculated (usually because PPV was 100%).

CSF NfL had a similar AUC: (0.89 [0.82, 0.96]). The optimal CSF cutoff of 823 pg/mL was associated with 95% specificity, 71% sensitivity, 15.71 LR+, 0.30 LR‐, 98% PPV, 54% NPV, 52.5 DOR, and 78% accuracy. An alternative CSF cutoff, optimizing for sensitivity was 531 pg/mL, 64% specificity, 95% sensitivity, 2.62 LR+, 0.07 LR‐, 88% PPV, 82% NPV, 35 DOR, and 87% accuracy. There was no statistical difference between plasma NfL and CSF performance (*p* = 0.520).

Considering specific ND subgroups that are the most common differential diagnoses, AD and bvFTD, plasma and CSF NfL had high diagnostic performance for all ages (AD vs. PPD AUCs: 0.89 [plasma] and 0.95 [CSF]; bvFTD vs. PPD AUCs: 0.79 [plasma], 0.86 [CSF]), with even stronger performance in younger people (full details in Table 2).

### 3.3 Aim 2: Age‐based cutoffs and cutoffs optimized for screening

We investigated cutoffs and diagnostic performance in younger people (40– < 60 years), and older people (60– < 70 years), similar to recent publications.^21^ We restricted to these age ranges as these had the greatest overlap between ND and PPD. In addition, we assessed alternative cutoffs optimized for screening, for 100%, 97.5%, 95%, and 90% specificity, and sensitivity, also similar to recent publications.^30^ Alternative cutoffs and additional information are presented in Table 2, Table S3 (which includes ratios of cutoffs), and Figures S1–S3.

#### 3.3.1 Younger people

In younger people (total 98, 48 PPD, 50 ND), plasma NfL had an AUC of 0.89 [0.83, 0.96], cutoff 14.6 pg/mL, 90% specificity, 84% sensitivity, 8.06 LR+, 0.18 LR‐, 89% PPV, 84% NPV, 45.2 DOR, 87% accuracy.

CSF NfL had an AUC of 0.97 [0.92, 1.00], 814 pg/mL cutoff, 100% specificity, 88% sensitivity, 0.13 LR‐, 100% PPV, 81% NPV, and 92% accuracy. An alternate cutoff, optimizing for sensitivity, was 558 pg/mL, 85% specificity, 96% sensitivity, 6.23 LR+, 0.05 LR‐, 92% PPV, 92% NPV, 126.5 DOR, and 92% accuracy. Plasma and CSF AUCs were not statistically different (*p* = 0.061).

#### 3.3.2 Older people

In older people (total 77, 20 PPD, 57 ND), plasma NfL had an AUC of 0.76 [0.63, 0.89], cutoff 11.9 pg/mL, 55% specificity, 91% sensitivity, 2.03 LR+, 0.16 LR‐, 85% PPV, 69% NPV, 12.7 DOR, 82% accuracy.

CSF NfL had an AUC of 0.76 [0.59, 0.92], 967 pg/mL cutoff, 100% specificity, 55% sensitivity, ∞ LR+, 0.45 LR‐, 100% PPV, 36% NPV, 64% accuracy. There were no differences in AUC between plasma and CSF (*p* = 0.961).

Comparing AUCs between younger and older groups, CSF NfL performed better in younger compared to older people (AUC: 0.97 vs. 0.76, *p* = 0.015), but while there was a trend, this was not the case for plasma NfL (0.89 vs. 0.76, *p* = 0.081).

### 3.4 Aim 3: Exploratory comparisons to large reference cohort, different classification systems, and their diagnostic performance

We explored a range of different classification systems, reference ranges and cutoffs, and their diagnostic utility, to understand these important factors and potential issues related to clinical translation.

#### 3.4.1 Comparing to large reference control cohort

We compared plasma NfL levels in our cohort to a previously described large reference control cohort (“Control Group 2”).^22^, ^31^

Plasma NfL levels in ND were significantly elevated when compared to Control Group 2, 25.2 pg/mL versus 8.34 pg/mL, ß: 0.36, 95% CI: [0.21, 1.41], *p* < 0.001). Interestingly, levels were also higher in PPD compared to Control Group 2 (PPD ß: 0.04 [0.01, 0.14], *p* = 0.004), and surprisingly, Controls compared to Control Group 2 as well (ß: 0.05 [0.02, 0.18], *p* < 0.001).

This was also reflected in NfL age‐adjusted *z*‐scores, derived from Control Group 2 as previously described.^22^ A large difference was seen between ND and Control Group 2 *z*‐scores (ß: 1.98 [1.81, 2.18], *p* < 0.001). Smaller differences were observed between Control Group 2 and PPD (ß: 0.57 [0.25, 1.07], *p* < 0.001) and Controls (ß: 0.44 [0.29, 0.57], *p* < 0.001).

To investigate the possibility of systematic and analytical bias, factors such as batch effect to explain these surprising findings, especially the differences between Controls and Control Group 2 (i.e., that plasma NfL levels from the batch analysis were systematically higher than the levels from the Control Group 2 batch), we looked at data from 19 samples from our cohort (Batch 1) that were subsequently also analyzed on a different batch (Batch 2), using the same analysis kit and platform. Levels between the two batches correlated strongly in a linear fashion (*R* ^2^ = 0.958), but levels in Batch 1 were on average approximately 1.4 times higher than levels in Batch 2.[Table alz14278-tbl-0002]

We then investigated the influence of this possible batch effect by applying a conversion formula and converting NfL levels in the present cohorts (Batch 1), adjusted level = 1.33 + 0.63 * x (see Table S4 and Figure S4). After applying this adjustment, plasma NfL levels were no longer different between PPD and Control Group 2 (ß: ‐0.02 [‐0.06, 0.02], *p* = 0.286), or between the present Controls and Control Group 2 (ß: 0.00 [‐0.05, 0.07], *p* = 0.810). *z*‐Scores derived from these adjusted NfL levels were also no longer different between PPD and Control Group 2 (*p* = 0.92) and between the present Controls and Control Group 2 (*p* = 0.176). ND remained elevated; however, compared to Control Group 2, even with adjusted levels (17.2 pg/mL vs. 8.34 pg/mL, ß: 0.27 [0.15, 0.79], *p* < 0.001), and the difference in z‐scores remained large (ß: 1.44 [1.20, 1.66], *p* < 0.001). This could suggest that (a) Batch 2 levels were closer to levels derived from Control Group 2 batches, and (b) batch‐to‐batch variability could result in potentially spurious findings, where there are small differences between groups.

While the plasma NfL diagnostic performance parameters described above (e.g., AUC, sensitivity, specificity, DOR) were unchanged when using adjusted plasma NfL levels, the values of optimal cutoff values were influenced. For example, the optimal plasma NfL cutoff for ND versus PPD for all ages changed from 14.1 pg/mL to 10.2 pg/mL when using the adjusted NfL levels.

For all ages, the diagnostic performance of *z*‐scores to distinguish ND from PPD was outperformed by plasma NfL (AUC: 0.80 vs. 0.86, *p* < 0.001 and CSF NfL (0.80 vs. 0.89, *p* = 0.037). In younger people, *z*‐scores were not statistically different from plasma NfL AUCs (0.87 vs. 0.89, *p* = 0.091), but were outperformed by CSF NfL (0.87 vs. 0.97, *p* = 0.031). In older people, there were no statistical differences in AUCs between plasma NfL and *z*‐scores (*p* = 0.856), or CSF NfL and *z*‐scores (*p* = 0.948). Using *z*‐scores based on adjusted NfL values did not result in improved AUCs (0.78 for all ages [vs. 0.80 unadjusted], 0.87 for younger [vs. 0.87], and 0.77 for older [vs. 0.77]).

#### 3.4.2 Compared to previously described cutoffs

We explored the diagnostic utility of other previously described plasma and CSF cutoffs.^3^, ^5^, ^31^

Age‐based plasma NfL cutoffs presented by Simrén et al.^31^ resulted in 73% specificity, 71% sensitivity, 2.59 LR+, 0.40 LR‐, 82% PPV, 58% NPV, 6.4 DOR, and 71% accuracy. The use of adjusted NfL levels (as described above) resulted in improved specificity: 90% specificity, 46% sensitivity, 4.39 LR+, 0.61 LR‐, 89% PPV, 48% NPV, 7.2 DOR, and 62% accuracy.

Using our previously described age‐based CSF NfL cutoffs^3^ resulted in 73% specificity, 83% sensitivity, 3.03 LR+, 0.24 LR‐, 90% PPV, 59% NPV, 12.6 DOR, 80% accuracy. Using only the 582 pg/mL cutoff across all ages previously described,^5^ resulted in 64% specificity, 94% sensitivity, 2.58 LR+, 0.10 LR‐, 88% PPV, 78% NPV, 25.8 DOR, and 86% accuracy. Once again, this improved in younger people: 85% specificity, 96% sensitivity, 6.23 LR+, 0.05 LR‐, 92% PPV, 92% NPV, 126.5 DOR, and 92% accuracy.

## 4 DISCUSSION

This study investigated the ability of plasma and CSF NfL to distinguish between diverse ND and PPD in patients referred to and assessed at a specialist clinic. We found significantly elevated plasma and CSF NfL levels in ND compared to PPD, and strong and comparable diagnostic performance of both plasma and CSF NfL to aid in this common, challenging clinical distinction. Diagnostic performance was especially high in younger people, and we described a range of cutoffs based on age and optimized for sensitivity and specificity. Finally, findings from exploratory analyses highlighted potential issues with sample analysis and choices of reference range, issues critical for future broad clinical implementation.

Strengths of this study included focusing on distinguishing diverse ND from a large group of diverse PPD, and the clinical and younger nature of the cohort. To our knowledge, other studies have not thus far investigated plasma NfL in distinguishing diverse ND from as large a group of well‐described PPD, all from a real‐world setting. We included a clinical cohort of patients, with no exclusion criteria, to ensure that the findings were reflective of a real‐world setting. Findings in diverse conditions and ages in a clinical setting provide important evidence for real‐world performance since current real‐world clinical practice involves broad differentials for people with symptoms rather than distinguishing ND from controls or distinguishing only AD from controls or preclinical AD. Understanding NfL levels in diverse PPD from clinical settings is important, since a significant proportion of people who present to clinical services with neuropsychiatric symptoms for assessment of a possible ND (especially younger‐onset) will be diagnosed with a PPD, and finally, since PPD may be associated with subtle abnormalities and cannot be assumed to be equivalent to or comparable to healthy controls.^21^, ^22^ Furthermore, we focused on a relatively younger cohort, in contrast to most previous studies in clinical settings, which have had older cohorts.^14^, ^16^, ^20^ This is an important group to investigate; the range of differential diagnoses and atypical presentations means that rates of misdiagnosis and diagnostic uncertainty are all higher in younger people compared to older people.^2^, ^32^, ^33^

Diagnostic performance and metrics of both plasma and CSF NfL were very high in younger people and were higher compared to older people, consistent with previous studies.^3^, ^21^ The AUC of CSF NfL in younger people was particularly high (AUC: 0.97). This difference was not statistically different from the AUC for plasma NfL in younger people (AUC: 0.89), suggesting that plasma NfL levels alone may be sufficient for diagnosis, and there may be little benefit in routinely adding on CSF NfL for a younger person who has already had their plasma NfL levels analyzed. Performance was weaker in older people for both plasma and CSF NfL; however, we still found the diagnostic utility of a single cutoff for NfL in older people, unlike other studies that did not.^21^

Our study results support using plasma NfL as a less invasive alternative to CSF NfL for differentiating ND from PPD, and using age‐based cutoffs optimized for sensitivity and sensitivity to aid interpretations. Levels above or below a single optimal cutoff would still offer strong diagnostic utility, especially in younger people; however, greater caution would be required for older people. For example, very high plasma NfL could indicate a neurodegenerative cause (and dismiss a primary psychiatric cause) of a patient's symptoms and facilitate the precision use of more specific investigations based on ND differential diagnoses (such as plasma ptau217 for AD).^34^, ^35^ Conversely, very low plasma NfL could indicate a primary psychiatric cause of symptoms. Although plasma NfL outperformed CSF NfL in terms of AUC, CSF NfL had very high DOR and accuracy for AD versus PPD and bvFTD versus PPD distinctions. Future research is needed to determine in what circumstances diagnosis would benefit from using both plasma and CSF NfL, such as when “borderline” plasma NfL levels are observed or there remains strong suspicion of ND.

Exploratory comparisons to a large reference cohort revealed some surprising findings, important for future research and clinical implementation. Variability of levels on different batches resulted in slightly higher levels in all groups in this study, compared to the reference cohort. This did not affect the utility of plasma NfL to distinguish ND from the reference cohort as NfL levels in ND were so highly elevated; however, this resulted in a possibly spurious finding of elevated levels in PPD (and the present control group) compared to the reference cohort. Adjusting our NfL levels to correct this batch effect reversed this finding of elevated levels in PPD and controls compared to the reference cohort, aligning with previous/expected results.^5^, ^22^ In addition, potential systematic batch or analysis factors would influence the actual value of the reference range or cutoff (for example, an optimal cutoff changed from 14.1 pg/mL to 10.2 pg/mL). These exploratory findings highlight potential limitations and the need for caution in using levels and reference ranges derived from other cohorts and different batches, caution in interpreting individual levels, especially levels relatively close to a reference range/cutoff or “borderline,” consistent with other studies that have investigated similar issues.^28^ Our findings support the importance of a local control group, contrary to our previous study where comparisons to the large reference cohort were comparable and at times were more useful than comparing to a local control group.^22^ In addition, the findings of this study would also suggest that raw levels and cutoffs were superior to our previously described age‐adjusted *z*‐scores, and age‐based cutoffs derived from the large reference cohort.^22^, ^31^ Future research should further investigate these potential issues, to advance analysis technologies and improve accurate interpretation of individual levels.

Limitations of this study include the relatively small subset of patients who had both plasma and CSF NfL, and the lack of serial NfL levels. Future studies will include larger numbers of people with both CSF and plasma NfL levels, and investigate diagnostic utility of serial NfL levels. An important issue is interpretation of initially ambiguous or “borderline” NfL levels, and the next steps for the clinician. Future research is required and underway to provide guidance in these situations, for example, on the utility of repeating plasma NfL levels as well as the optimal delay between collections, and the additional value of CSF NfL. In addition, several covariate or confounding variables are known to acutely/subacutely elevate NfL levels for several weeks to months (such as recent head injury, stroke, delirium).^36^, ^37^, ^38^, ^39^, ^40^, ^41^ Further research including such patients is required, to properly understand the diagnostic performance and delay to testing for screening for neurodegeneration in these scenarios. Most patients in this study did not have definitive confirmation, such as genetic or postmortem confirmation of ND. While patients had comprehensive multidisciplinary assessments and multimodal investigations with current gold standard clinical diagnosis and longitudinal follow up, the limitations and instability of even gold standard clinical diagnosis are recognised.^2^, ^3^, ^7^ Of note, CSF NfL in this study's subset of 85 patients had a slightly lower AUC for all ages compared to our previous study (0.89 vs. 0.94), which had a larger sample size and longer follow up duration.^5^ The smaller sample sizes in this study could also have contributed to wider CIs and difficulty detecting true differences between AUCs. Therefore, it is possible that with larger sample sizes, additional time and follow up of patients, the diagnostic categorization and overall findings from this study for both plasma and CSF NfL could be different. The relatively small sample of older people, especially people over age 70, means that our findings in older people must be taken with caution, and replicated in larger studies. While the ND group included the most common types of dementia such as AD, bvFTD, DLB, due to the specialized nature of our clinical service that includes a Huntington's disease service, it also included patients with these rarer causes.^1^, ^2^, ^42^ Finally, our findings from a specialist service cannot be directly translated to lower prevalence settings, such as primary care, and studies are underway in these settings.

To conclude, this study found strong diagnostic performance of both plasma and CSF NfL to distinguish ND from PPD in a real‐world clinical setting. NfL had particularly strong diagnostic performance in younger people, where the range of differential diagnoses and atypical presentations, misdiagnosis, and diagnostic delay, are all greater.^2^, ^32^, ^33^ The comparability of plasma NfL to CSF NfL adds important evidence for the utility of a simple blood‐based biomarker to assist in a common, yet challenging clinical situation, as a screening test for neurodegeneration, akin to a “CRP for the brain,” to reduce misdiagnosis and delay and improve precision care and outcomes for patients, their families, and healthcare systems. Future research will need to focus on implementation and translational issues such as analytical, technological, and reference range issues.

## CONFLICT OF INTEREST STATEMENT

H.Z. has served at scientific advisory boards and/or as a consultant for Abbvie, Acumen, Alector, Alzinova, ALZPath, Amylyx, Annexon, Apellis, Artery Therapeutics, AZTherapies, Cognito Therapeutics, CogRx, Denali, Eisai, LabCorp, Merry Life, Nervgen, Novo Nordisk, Optoceutics, Passage Bio, Pinteon Therapeutics, Prothena, Red Abbey Labs, reMYND, Roche, Samumed, Siemens Healthineers, Triplet Therapeutics, and Wave, has given lectures in symposia sponsored by Alzecure, Biogen, Cellectricon, Fujirebio, Lilly, Novo Nordisk, and Roche, and is a co‐founder of Brain Biomarker Solutions in Gothenburg AB (BBS), which is a part of the GU Ventures Incubator Program (outside submitted work). S.L. has received an honorarium from Lundbeck and previously received NHMRC funding. T.K. served on scientific advisory boards for MS International Federation and World Health Organisation, BMS, Roche, Janssen, Sanofi Genzyme, Novartis, Merck, and Biogen, the steering committee for Brain Atrophy Initiative by Sanofi Genzyme received conference travel support and/or speaker honoraria from WebMD Global, Eisai, Novartis, Biogen, Roche, Sanofi‐Genzyme, Teva, BioCSL and Merck and received research or educational event support from Biogen, Novartis, Genzyme, Roche, Celgene and Merck. K.B. has served as a consultant and on advisory boards for Abbvie, AC Immune, ALZPath, AriBio, BioArctic, Biogen, Eisai, Lilly, and Moleac Pte. Ltd, Neurimmune, Novartis, Ono Pharma, Prothena, Roche Diagnostics, and Siemens Healthineers; has served on data monitoring committees for Julius Clinical and Novartis; has given lectures, produced educational materials and participated in educational programs for AC Immune, Biogen, Celdara Medical, Eisai and Roche Diagnostics; and is a co‐founder of Brain Biomarker Solutions in Gothenburg AB (BBS), which is a part of the GU Ventures Incubator Program, outside the work presented in this paper. K.B. is supported by the Swedish Research Council (#2017‐00915 and #2022‐00732), the Swedish Alzheimer Foundation (#AF‐930351, #AF‐939721, #AF‐968270, and #AF‐994551), Hjärnfonden, Sweden (#FO2017‐0243 and #ALZ2022‐0006), the Swedish state under the agreement between the Swedish government and the County Councils, the ALF‐agreement (#ALFGBG‐715986 and #ALFGBG‐965240), the European Union Joint Program for Neurodegenerative Disorders (JPND2019‐466‐236), the Alzheimer's Association 2021 Zenith Award (ZEN‐21‐848495), the Alzheimer's Association 2022‐2025 Grant (SG‐23‐1038904 QC), La Fondation Recherche Alzheimer (FRA), Paris, France, the Kirsten and Freddy Johansen Foundation, Copenhagen, Denmark, and Familjen Rönströms Stiftelse, Stockholm, Sweden. This study was supported by funding from NHMRC (1185180), MACH MRFF RART 2.2, CJDSGN Memorial Award in memory of Michael Luscombe and RMH Foundation Spring Appeal. All the other authors have nothing to disclose. Author disclosures are available in the Supporting Information.

## CONSENT STATEMENT

All human subjects provided informed consent.

## Supporting information



Supporting Information

Supporting Information

## 1 COLLABORATORS/CONTRIBUTORS

On behalf of others in The MiND Study Group:

**Table:**

Name	Affiliations (separated by semi‐colon)
Hannah Dobson	Psychiatry, Alfred Health; Neuropsychiatry, Royal Melbourne Hospital
Rosie Watson	Population Health and Immunity Division, The Walter and Eliza Hall Institute of Medical Research, Parkville, Australia;
	Department of Aged Care and Medicine, The Royal Melbourne Hospital, University of Melbourne, Parkville, Australia
Nawaf Yassi	Population Health and Immunity Division, The Walter and Eliza Hall Institute of Medical Research, Parkville, Australia;
	Department of Medicine and Neurology, Melbourne Brain Centre at the Royal Melbourne Hospital, University of Melbourne, Parkville, Australia
Terence J. O'Brien	Department of Neuroscience, The Central Clinical School, Monash University
Patrick Kwan	Department of Neuroscience, The Central Clinical School, Monash University
Christos Pantelis	Melbourne Neuropsychiatry Centre & Department of Psychiatry, University of Melbourne & Melbourne Health; Mid‐West Area Mental Health Service, Melbourne Health
Oskar Hansson	Department of Clinical Sciences, Clinical Memory Research Unit, Faculty of Medicine, Lund University, Lund/Malmö, Sweden
Shorena Janelidze	Department of Clinical Sciences, Clinical Memory Research Unit, Faculty of Medicine, Lund University, Lund/Malmö, Sweden
Christopher Fowler	The Florey Institute of Neuroscience and Mental Health, The University of Melbourne, Parkville, VIC
Richard Kanaan	Dept of Psychiatry, University of Melbourne, Austin Health, Heidelberg, VIC 3084
Piero Perucca	Department of Medicine, Austin Health, The University of Melbourne; Comprehensive Epilepsy Program, Austin Health; Department of Neuroscience, Central Clinical School, Monash University; Department of Neurology, The Royal Melbourne Hospital; Department of Neurology, Alfred Health, Melbourne, VIC, Australia
Anna King	The Wicking Dementia Centre, Tasmania
Jane Gunn	Department of General Practice, The University of Melbourne
Tianxin Pan	Health Economics Unit | Centre for Health Policy | Melbourne School of Population and Global Health, The University of Melbourne
Ilias Goranitis	Health Economics Unit | Centre for Health Policy | Melbourne School of Population and Global Health, The University of Melbourne
Steve Simpson‐Yap	CORe, The Royal Melbourne Hospital, The University of Melbourne, Melbourne, VIC, Australia
