# Supplementary material for: Plasma and CSF neurofilament light chain distinguish neurodegenerative from primary psychiatric conditions in a clinical setting
Source: Alzheimers Dement. 2024 Oct 6;20(11):7989–8001. doi: 10.1002/alz.14278 (PMC11567869; doi:10.1002/alz.14278)
Supplement: Supplementary file 1 — Supporting Information [file ALZ-20-7989-s002.pdf]

SUPPLEMENTARY MATERIAL

	ND	PPD	N
	N=63	*N=22*	
age	60.9 [57.4;65.0]	58.6 [51.3;63.7]	85
sex: Female	27 (42.9%)	7 (31.8%)	85
weight	74.2 [63.2;91.7]	82.0 [79.0;86.1]	67
nfl	24.4 [15.6;35.8]	10.3 [7.55;12.3]	85
lognfl	1.39 [1.19;1.55]	1.01 [0.88;1.09]	85
nfl.z	2.05 [1.44;2.69]	0.15 [-0.54;1.25]	85
nfl.percentile	0.98 [0.92;1.00]	0.56 [0.30;0.89]	85
nfl_csf_pilot2024	1048 [741;1585]	495 [386;671]	85
logcsfnfl	3.02 [2.87;3.20]	2.69 [2.59;2.83]	85

Supplementary Table 1. Subset with paired CSF and plasma
Data are median [interquartile range] or n (%).

	ND	PPD	Control	N
	N=73	*N=55*	*N=124*	
age	60.8 [52.8;67.8]	54.3 [43.7;59.4]	63.2 [56.0;70.0]	252
sex: Female	32 (43.8%)	32 (58.2%)	88 (71.0%)	252
weight	75.8 [58.4;86.0]	87.0 [71.5;100]	76.0 [66.0;84.0]	183
nfl	26.8 [16.6;43.0]	10.1 [8.05;12.8]	12.5 [8.70;17.8]	252
lognfl	1.43 [1.22;1.63]	1.00 [0.91;1.11]	1.10 [0.94;1.25]	252
nfl.z	2.36 [1.34;3.12]	0.70 [-0.01;1.53]	0.47 [-0.13;1.24]	252
nfl.percentile	0.99 [0.91;1.00]	0.76 [0.50;0.94]	0.68 [0.45;0.89]	252
nfl_csf_pilot2024	.	.	.	0
logcsfnfl	.	.	.	0

Supplementary Table 2. Subset with plasma and no CSF
Data are median [interquartile range] or n (%).

Categorisation	Age	AUC	Cutoff	Cutoff ratio ^^	Spec	Sens
**ND vs PPD**						
Plasma NfL	All	0.86 [0.81, 0.92]	14.1	1.40	81%	85%
CSF NfL	All	0.89 [0.82, 0.96]	823 (532)	1.66 (1.07)	95% (64%)	71% (95%)
Plasma NfL	Younger 40-<60	0.89 [0.83, 0.96]	14.6 [a]	1.45	90%	84%
CSF NfL	Younger 40-<60	0.97 [0.92, 1]	814 [b] (558)	1.88 (1.29)	100% (85%)	88% (96%)
Plasma NfL	Older 60-<70	0.76 [0.63, 0.89]	11.9 [c]	1.03	55%	91%
CSF NfL	Older 60-<70	0.76 [0.59, 0.92]	967 [d]	1.36	100%	55%
**AD vs PPD [e]**						
Plasma NfL	All	0.89 [0.84, 0.95]	14.6	1.45	82%	93%
CSF NfL	All	0.95 [0.90, 1]	824	1.66	95%	87%
**bvFTD vs PPD [f]**						
Plasma NfL	All	0.79 [0.65, 0.92]	11.9	1.18	69%	81%
CSF NfL	All	0.86 [0.70, 1]	975	1.97	100%	63%

Supplementary Table 3. Details of ROC curve analyses and diagnostic test parameters, including cut-off expressed as a ratio

AD: Alzheimer disease; AUC: area under the curve; bvFTD: behavioural variant frontotemporal dementia; CSF: cerebrospinal fluid; LR+: positive likelihood ratio; LR-: negative likelihood ratio; NfL: neurofilament light chain; ND: neurodegenerative disorder; NPV: negative predictive value; PPD: primary psychiatric disorder; PPV: positive predictive value; Sens: sensitivity; Spec: specificity

^^: The cut-off ratio was calculated based on a ratio of the optimal cut-off and the median NfL level of the PPD group for each categorisation/comparison

a: Alternative cut-offs optimising specificity were: 30.8pg/mL (100% specificity), 24.4pg/mL (98% specificity), 24pg/mL (96% specificity), 14.6pg/mL (90%), and for sensitivity: 6.02pg/mL (100%), 8.04pg/mL (98%), 10.1pg/mL (94%), 11.5pg/mL (90%).

b: Alternative CSF cutoffs for specificity were 814pg/mL (100% specificity), 743pg/mL (92%), 558pg/mL (85%), and for sensitivity were 445pg/mL (100% sensitivity), 558pg/mL (96% sensitivity, 85% specificity), 638pg/mL (92%).

c: Alternative cut-offs associated with 100%, 95%, 90% specificity were 74.9pg/mL, 31.8pg/mL, 31pg/mL, respectively. Alternative cut-offs optimising for sensitivity were 7.59pg/mL (100% sensitivity), 10.35pg/mL (98%), 10.85pg/mL (95%), 11.9pg/mL (91%).

d: Alternative cut-offs optimising for specificity were 967pg/mL (100%) and 823pg/mL (88%), and for sensitivity were 511pg/mL (97%), 571pg/mL (94%), 600pg/mL (90%).

e: Higher DOR and accuracy in younger people (154 and infinity, 91% and 96%, for plasma and CSF NfL respectively)

f: Higher DOR and accuracy in younger people (16.5 and infinity, 86% and 94%, for plasma and CSF NfL respectively)

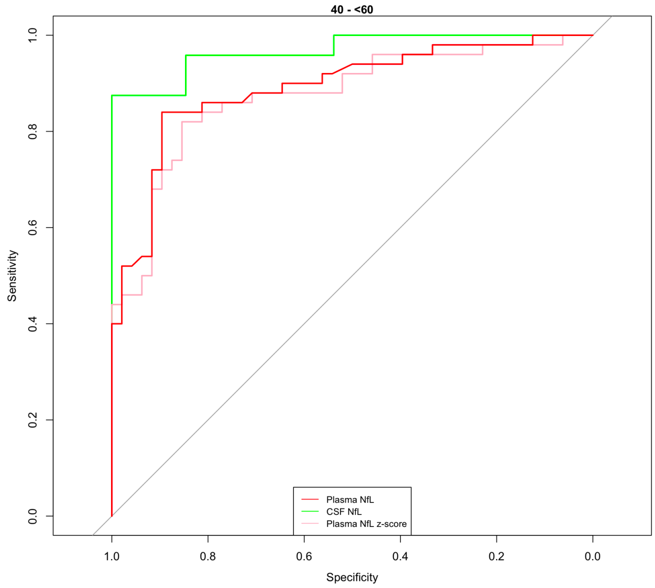

Supplementary Figure 1. ROC analysis for younger people (40 - <60yo)

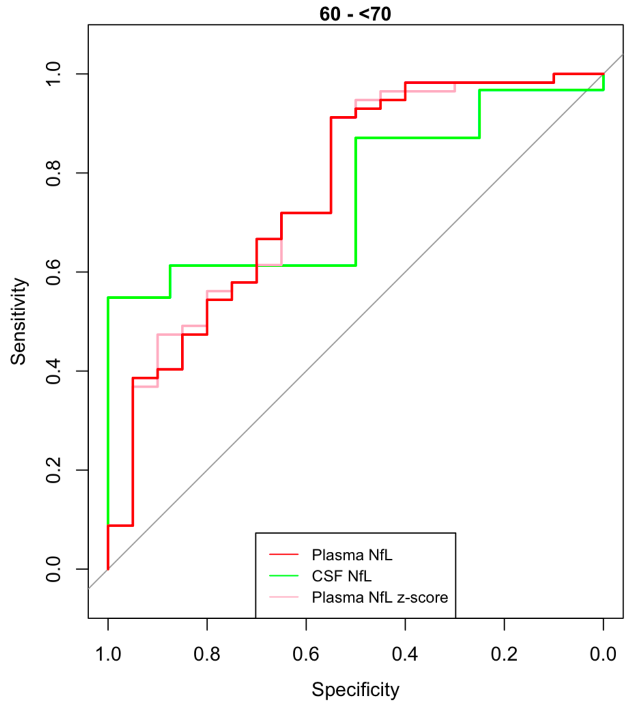

Supplementary Figure 2. ROC analysis for older people 60 - <70

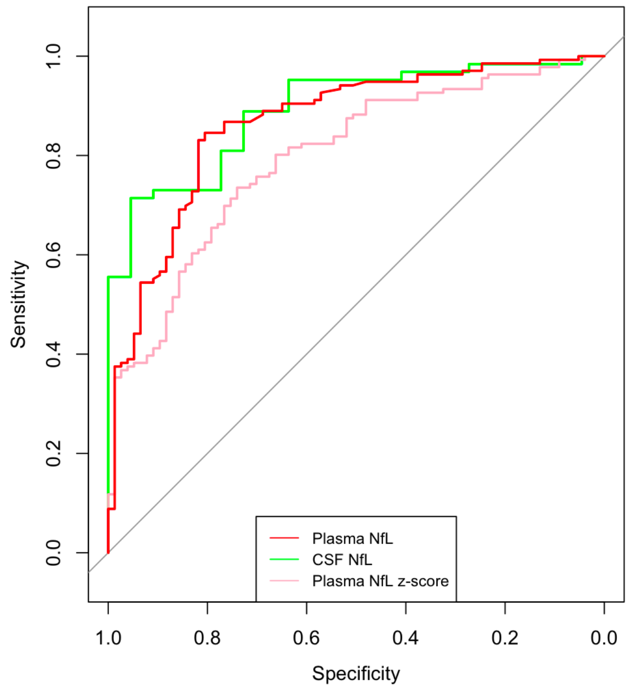

Supplementary Figure 3. ROC analysis for all ages including z-scores

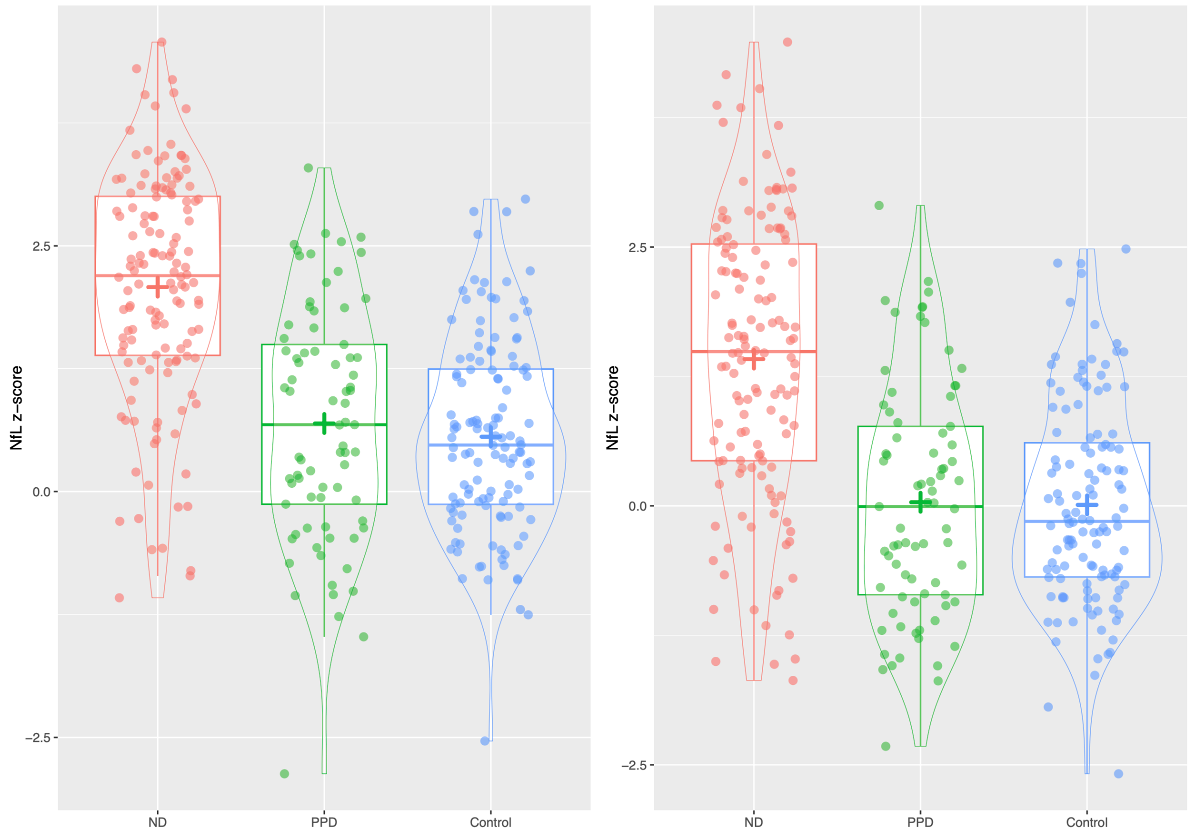

Supplementary Figure 4. Z-scores before adjustment/conversion (left) and after adjustment/conversion (right).

	ND	PPD	Control	Control Group 2	N
	N=136	*N=77*	*N=124*	*N=1926*	
age	60.8 [55.9;65.9]	54.8 [46.6;61.8]	63.2 [56.0;70.0]	56.0 [48.0;64.0]	2263
sex: Female	59 (43.4%)	39 (50.6%)	88 (71.0%)	1218 (63.2%)	2263
weight	75.0 [59.8;89.1]	84.0 [73.4;98.8]	76.0 [66.0;84.0]	. [.;.]	250
nfl	25.2 [15.8;39.6]	10.1 [7.85;12.5]	12.5 [8.70;17.8]	8.34 [6.10;11.6]	2263
lognfl	1.40 [1.20;1.60]	1.00 [0.89;1.10]	1.10 [0.94;1.25]	0.92 [0.79;1.06]	2263
nfl.z	2.20 [1.39;3.00]	0.68 [-0.13;1.50]	0.47 [-0.13;1.24]	-0.01 [-0.70;0.71]	2263
nfl.percentile	0.99 [0.92;1.00]	0.75 [0.45;0.93]	0.68 [0.45;0.89]	0.50 [0.24;0.76]	2263
nfl.adjusted	17.2 [11.3;26.3]	7.69 [6.27;9.20]	9.93 [7.77;14.3]	8.34 [6.10;11.6]	2263
lognfl.adjusted	1.24 [1.05;1.42]	0.89 [0.80;0.96]	1.00 [0.89;1.16]	0.92 [0.79;1.06]	2263
nfl.adjusted.z	1.49 [0.44;2.53]	-0.01 [-0.86;0.77]	-0.15 [-0.69;0.61]	-0.01 [-0.70;0.71]	2263
nfl.adjusted.percentile	0.93 [0.67;0.99]	0.50 [0.20;0.78]	0.44 [0.25;0.73]	0.50 [0.24;0.76]	2263

Supplementary Table 4. Adjusted plasma NfL levels and details of Control Group 2
